# New Evidence of Gut Microbiota Involvement in the Neuropathogenesis of Bipolar Depression by *TRANK1* Modulation: Joint Clinical and Animal Data

**DOI:** 10.3389/fimmu.2021.789647

**Published:** 2021-12-21

**Authors:** Jianbo Lai, Peifen Zhang, Jiajun Jiang, Tingting Mou, Yifan Li, Caixi Xi, Lingling Wu, Xingle Gao, Danhua Zhang, Yiqing Chen, Huimin Huang, Huijuan Li, Xin Cai, Ming Li, Peng Zheng, Shaohua Hu

**Affiliations:** ^1^ Department of Psychiatry, The First Affiliated Hospital, Zhejiang University School of Medicine, Hangzhou, China; ^2^ The Key Laboratory of Mental Disorder’s Management in Zhejiang Province, Hangzhou, China; ^3^ Brain Research Institute of Zhejiang University, Hangzhou, China; ^4^ MOE Frontier Science Center for Brain Science & Brain-Machine Integration, Zhejiang University, Hangzhou, China; ^5^ Department of Neurology, The First Affiliated Hospital of Chongqing Medical University, Chongqing, China; ^6^ NHC Key Laboratory of Diagnosis and Treatment on Brain Functional Diseases, The First Affiliated Hospital of Chongqing Medical University, Chongqing, China; ^7^ Institute of Psychiatry, Wenzhou Medical University, Wenzhou, China; ^8^ Key Laboratory of Animal Models and Human Disease Mechanisms of the Chinese Academy of Sciences and Yunnan Province, Kunming Institute of Zoology, Chinese Academy of Sciences, Kunming, China

**Keywords:** bipolar disorder, gut microbiota, *TRANK1*, neuroinflammation, fecal microbiota transplantation

## Abstract

Tetratricopeptide repeat and ankyrin repeat containing 1 (*TRANK1*) is a robust risk gene of bipolar disorder (BD). However, little is known on the role of *TRANK1* in the pathogenesis of BD and whether the gut microbiota is capable of regulating *TRANK1* expression. In this study, we first investigated the serum mRNA level of *TRANK1* in medication-free patients with a depressive episode of BD, then a mice model was constructed by fecal microbiota transplantation (FMT) to explore the effects of gut microbiota on brain *TRANK1* expression and neuroinflammation, which was further verified by *in vitro* Lipopolysaccharide (LPS) treatment in BV-2 microglial cells and neurons. 22 patients with a depressive episode and 28 healthy individuals were recruited. Serum level of *TRANK1* mRNA was higher in depressed patients than that of healthy controls. Mice harboring ‘BD microbiota’ following FMT presented depression-like phenotype. mRNA levels of inflammatory cytokines and *TRANK1* were elevated in mice hippocampus and prefrontal cortex. *In vitro*, LPS treatment activated the secretion of pro-inflammatory factors in BV-2 cells, which was capable of upregulating the neuronal expression of *TRANK1* mRNA. Moreover, primary cortical neurons transfected with plasmid Cytomegalovirus DNA (pcDNA3.1(+)) vector encoding human *TRANK1* showed decreased dendritic spine density. Together, these findings add new evidence to the microbiota-gut-brain regulation in BD, indicating that microbiota is possibly involved in the neuropathogenesis of BD by modulating the expression of *TRANK1*.

## Introduction

Bipolar disorder (BD) is a recurrent, debilitating mood disorder with a high heritability (1). Although the exact etiology of BD is sophisticated, the dominant role of both gene and environmental factors on the onset and development of BD is widely accepted (1, 2). With the technological advances in molecular biology, susceptible genes related to BD have been increasingly identified (3, 4). For example, several genome-wide association studies (GWAS) and subsequent independent verifications have indicated that tetratricopeptide repeat and ankyrin repeat containing 1 (*TRANK1*), located on the short arm of chromosome 3 (3p22.2), is a robust risk gene of BD (3–6). In postmortem brains of BD subjects, the expression of *TRANK1* was elevated compared to that of healthy individuals (7). TRANK1 protein is secreted mainly by immunocytes and is widely distributed in body tissues, including hippocampus, amygdala and other brain regions. Although the implications of *TRANK1* gene in BD remained largely unclear, a newly published large-scale analyses of mRNA co-expression network revealed that genes closely interact with *TRANK1*, such as glycogen synthase kinase-3 (GSK-3α/β), were engaged in the modulation of synaptic plasticity, neural growth, as well as circadian rhythm (6). More recently, an up-stream regulatory role of GSK-3α/β on *TRANK1* transcription has been unraveled (8), partially *via* the activation the transcription factor, CCAAT/enhancer-binding protein-α (C/EBP-α). Blockage of GSK-3α/β pathways or direct suppression of GSK-3α/β expression significantly led to reduced expression of *TRANK1* in U-251 human glioblastoma cells (8). Therefore, further investigations on the interactome of *TRANK1* with other genetic and environmental factors relevant to BD help to uncover the pathogenesis of this intractable psychiatric disorder.

Recently, microbiota inhabiting in the human gastrointestinal tract has been recognized as a pivotal environmental factor in regulating physical and mental well-being (9, 10). In previous studies, we have characterized the alterations in diversity and compositions of gut microbiota in patients with a depressive episode of BD when compared to healthy individuals (11, 12). Classification models derived from specific bacterial species not only had the potential to distinguish bipolar depression from health individuals or unipolar depression (11, 12), but also was capable of predicting the efficacy of mood stabilizing treatment (11). Notably, we found that compared to healthy individuals, most bacteria enriched in patients with bipolar depression belonged to the *Bacteroides* and *Flavobacterium* (11), both of which were gram-negative bacterial family that could produce lipopolysaccharide (LPS). Interestingly, a negative correlation between gut microbial diversity and methylation of the aryl hydrocarbon receptor nuclear translocator-like gene (*ARNTL*), a molecular clock gene linked to circadian rhythm, was revealed in BD patients (13). In our recent review, we have systematically discussed a hypothesis that gut microbe-derived LPS could influence the host *TRNAK1* expression in BD (14). These preliminary findings indicate the involvement of the microbiota-gut-brain regulation in BD. However, currently available studies on gut microbiota in BD were mainly genetically sequencing-based, or performed correlation analyses between indices of gut microbiota and clinical parameters, thus precluding the further interpretations of findings. Also, animal models focusing on the mechanistic pathways linking the microbiota to the brain are absent in BD studies.

The microbiota-gut-brain axis is known to operate in a bidirectional regulation pattern, which mainly consists of the metabolic, immune, endocrine and automatic nerves pathways (15). Notably, a neuroinflammatory basis underlying the development and progression of BD has been recognized recently (16, 17). Proinflammatory mediators in the peripheral, such as interleukin-1β (IL-1β), IL-6, and tumor necrosis factor-α (TNF-α), as well as neuroinflammatory markers in the central nervous system (activated microglia), are found to be elevated not only in the acute mood episodes, but also in the remission phase of BD (16, 18). In previous studies, we also found abnormal expression of immune checkpoint inhibitors on peripheral CD8+T cells, such as T cell immunoglobulin and mucin domain 3 (TIM-3), indicating potential disturbances in cellular immunity in BD individuals (19, 20). Moreover, the neuroinflammatory modifications may interact with or be affected by the unbalanced kynurenine pathways and reduction in the expression of neurotrophic factors, such as brain-derived neurotrophic factor (BDNF) (16, 21). Kynurenine aminotransferase-2 (KAT-2) is the key role enzyme regulating the production of neuroprotective kynurenic acid (KYNA) from kynurenine (KYN) in the tryptophan metabolism (22). Intriguingly, a most recently published study provided robust evidence that microbial biomolecules from the intestinal tract could be transferred to brain and other organs *via* outer membrane vesicles (23). Based on these findings, we speculate that the regulatory role of gut microbiota on host genes in BD may link to a complicated neuroinflammatory mechanism.

In this study, we first explored the serum expression of *TRANK1* mRNA in patients with BD depression, then constructed a mice model *via* fecal microbiota transplantation (FMT) to investigate the impact of gut microbiota on *TRANK1* expression and neuroinflammation. We further *in vitro* examined the effects of LPS treatment on microglia, and inflammatory stimuli on the *TRANK1* mRNA expression in neurons. The impact of *TRANK1* overexpression on the morphology of neurons was also investigated *via* transfection with plasmid Cytomegalovirus DNA (pcDNA3.1(+)) vector encoding human *TRANK1*. Collectively, this study helps to reveal that gut microbiota may participate in the BD pathogenesis by interacting with a robust BD risk gene.

## Methods

In accordance with the Helsinki Declaration, this study was approved by the Institutional Review Board of the First Affiliated Hospital, Zhejiang University School of Medicine (#2017-397). Written informed consent was obtained from all participants before enrollment.

### Participants

22 patients meeting the criteria for a depressive episode of bipolar disorder according to the *DSM-IV-TR* were recruited from the Psychiatry Department of our hospital. The diagnosis was further confirmed by an experienced clinical psychiatrist using the Mini International Neuropsychiatric Interview (24). 17-item Hamilton Depression Rating Scale (HDRS-17) (25) as used for assessing the severity of depression and Young Mania Rating Scale (YMRS) (26) for assessing mania. In our study, an HDRS-17 score ≥ 14 was considered as a current depressive episode. All BD candidates were required to be treatment-naive or drug-free for ≥ 3 months and had no comorbidity with any other psychiatric disorder. 28 healthy individuals (HCs) without a history of any psychiatric disorder were recruited. Exclusion criteria for all subjects included: a) acute or chronic infection; b) autoimmune diseases or other systematic diseases associated with immune dysregulation; c) severe physical diseases (e.g., cancer, diabetes); d) consumption of prebiotics, probiotics or antibiotics within 1 month prior to recruitment; e) pregnant or lactating females or in menstruation.

### Peripheral Expression of *TRANK1*

Peripheral venous blood (1ml) was collected from all participants in a fasting state at 7:00~8:00 a.m. and immediately used for detecting mRNA expression. Total RNA from human blood was isolated using Spin Column Blood Total RNA Purification Kit according to the manufacturer’s instruction (Sangon Biotech; Order No. B518653). Reverse transcription was performed using 4×EZscript Reverse Transcription Mix II (EZBioscience; Cat. No.EZB-RT2GQ) in a 20 ul reaction volume. *TRANK1* mRNA was detected by QuantStudio 5DX Real-Time PCR System using SYBR Green Fast qPCR Mix (ABclonal, Wuhan, China). Each sample was tested three times and GAPDH was used as an internal control. Forward primer (5’-3’) for TRANK1 (human) was CAGCACTCCACATCTTTCTAGA and reverse primer (5’-3’) was TTGAGGTAGTCGAATTCAGTGG.

### FMT and Behavioral Tests

To determine whether gut microbiota from patients with a depressive episode of BD was sufficient to cause depression-like behaviors in mice, FMT from HCs and untreated patients were performed. The animal experiment protocol was approved by the Hospital Animal Ethical Committee (Approval No. #2019-6).

#### FMT Procedures

At baseline, fecal samples were collected from all participants. Fecal samples from untreated patients with a depressive episode of BD (n = 10, age 16–43 years) and HCs (n = 10, age 16–40 years) were randomly chosen to colonize the guts of mice. The demographic and clinical profiles of these participants were shown in **Supplemental Table 1**. Fecal samples for FMT were handled under anaerobic conditions and detailed procedures for preparing feces were previously described.^[81]^ Each fecal sample (0.1 g) was suspended with 1.5 ml of reduced sterile phosphate-buffered saline, and pools were made from equal volumes of donor suspensions. Adult (6-8-week-old) male Kunming mice were colonized with pooled samples derived from either BD patients or HCs. Different groups of recipient mice were separately bred in different gnotobiotic isolators to prevent contamination of gut microbiota. Within each individual gnotobiotic isolator, either ‘BD microbiota’ or ‘healthy microbiota’ recipient mice were bred in different cages (five mice per cage). Mice were weighted at the beginning of FMT experimentation and immediately prior to sacrifice of the mice. The behavioral tests (including OFT and FST) were performed on week 1 and 2 after fecal transplantation. Brain samples were collected immediately when the mice were sacrificed and snap-frozen in liquid N_2_ and stored at -80°C.

#### Mice Behavioral Tests

Before initiation of the experiments, mice were kept in flexible film gnotobiotic isolators. Mice were fed the same chow and water with autoclaved treatment under a 12-h light-dark cycle (lights on at 07:30 AM), a constant temperature of 21-22°C and humidity of 50-60%. Before each behavioral test, mice were transferred to the specialized experimental room for acclimation ≥ 1 h prior to the test. Experimenters who carried out these tests were blind to the animal groupings between 08:00 and 17:00. A video-computerized tracking system (SMART, Panlab, Barcelona, Spain) was used to videotape and quantify the process of behavioral tests.

#### Open-Field Test (OFT) and Forced Swimming Test (FST)

The detailed procedures for OFT and FST were previously described.^[13]^ In OFT, the total motion distance was considered to reflect the degree of locomotor activity, while the proportion of distance spent in the center (inner 25% part of the total surface area) was considered as an index of anxiety-like behavior. In FST, immobility was defined as the absence of all motion with the exception of movements to keep the mouse’s head over water surface. Test sessions lasted for 6 minutes and the latter 5 minutes scored for immobility, which was considered as a proxy of depression-like behavior.

### Expression of Molecules of Interest in Mice Brain Following FMT

Total RNAs from mice brain tissues were isolated using Trizol reagent according to the manufacturer’s instructions (Invitrogen, USA). Reverse transcription was performed using an ABScript II cDNA First Strand Synthesis Kit (ABclonal, Wuhan, China) in a 20 ml reaction volume. The genes of interest were detected by QuantStudio 5DXReal-Time PCR System using SYBR Green Fast qPCR Mix (ABclonal, Wuhan, China). Each sample was tested three times and GAPDH was used as an internal control. Primers for GAPDH, IL-1β, IL-6, IFN-1β, TIM-3, KAT-2, TRANK1 and BDNF are listed in **Supplemental Table 2**.

### Effects of LPS Treatment on the *TRANK1* Expression in Neurons

#### BV-2 culture and Treatment

The mice BV-2 cell line (Invitrogen, USA) was cultured in six-well plates at 37°C with 5% CO_2_ incubator, supplemented by high glucose DMEM medium with 10% FBS. BV-2 were stimulated by LPS (100ng/ml) for 24h when cells proliferated to 70~80%. Then, cell supernatant was collected for stimulating primary CNS neuron, and BV-2 were used to extract RNA for examining inflammatory cytokines levels (IL-1β, IL-6 and TNF-α).

#### CNS Neuron Cultures and Treatment

Sprague Dawley rats were anesthetized and euthanized at E18 days. Hippocampus of fetal rats was immediately dissected and digested by trypsin into a single-cell suspension. Hippocampus neurons were counted (0.02*10^6^ cells/cm^2^) and seeded in six-well plates coated with poly-D-lysine (10 μg/mL), supplemented by neuro-basal medium with 2% B27 (Invitrogen, USA), 2.0 mM glutamax and 2.5% FBS. The neurons were cultured at 37°C with 5% CO_2_ to day 21 and then were stimulated by the previously LPS-treated BV-2 suspension for 24h. *TRANK1* gene expression in hippocampus neurons after treatment were measured by RT-PCR.

#### RT-PCR

The steps of RT-PCR in BV-2 cells and hippocampus neurons were in accordance with the section “Expression of molecules of interest in mice brain following FMT”. Each sample was tested three times and β-actin was used as an internal control. Primers for IL-1β, IL-6, TNF-α and TRANK1 are given in **Supplemental Table 3**.

### *TRANK1* Overexpression in Primary Cortical Neurons

#### TRANK1 Transfection

To determine the impact of *TRANK1* overexpression on neuronal morphology, pcDNA3.1(+) vector encoding human *TRANK1* with a C-terminus FLAG-tag was constructed. Integrity of the recombinant was verified by Sanger sequencing. Given that signals of FLAG fluorophore alone failed to provide ideal resolution for analyzing dendritic spine structures under our experimental condition, the Venus vector encoding the EGFP protein was co-transfected in all groups. Briefly, the recombinant constructs for *TRANK1* or control vectors (i.e., empty pcDNA3.1) were respectively transfected into rat neurons together with Venus at days *in vitro* (DIV) 14 using Lipofectamine 3000 (Life Technologies) according to the manufacturer’s protocol. Confocal analyses were performed 72 hours after the transfection.

#### Neuronal Morphology Analysis

The transfected neurons were fixed with PBS (4% paraformaldehyde plus 4% sucrose) at room temperature and stained with antibodies against FLAG (Rabbit monoclonal FLAG antibody, CST, #14793S) and GFP (Chicken polyclonal GFP antibody, Abcam, #ab13970). Fluorescence positive neurons were randomly selected for images captured with an LSM 880 Basic Operation (Carl Zeiss) using consistent acquisition parameters. The dendritic spine analyses were then carried out as previously described. In brief, Neuron Studio was used to semi-automatically analyze spines on secondary and tertiary dendrites. Each experimental group contained at least 15 neurons with satisfactory demonstration of at least two dendrites for averaged analyses.

### Statistical Analysis

Clinical and experimental data generated in this study were analyzed with SPSS 20.0 Statistical Package (IBM, IL, USA). Categorical data was conducted with chi-square test, while measurement data was calculated with independent sample t-test (two-tailed). To analyze the neuronal morphology following *TRANK1* transfection, two-tailed t-test and two-way ANOVA (multiple comparisons using Fisher’s LSD) were conducted to calculate the statistical differences between two experimental conditions. *P* < 0.05 was set as statistically significant.

## Results

### Demographic and Clinical Characteristics of Participants

22 patients with a depressive episode of BD and 28 HCs were included in this study. No significant difference was found in age, sex or BMI between these two groups (all *P* > 0.05). BD patients scored averagely at 26.64 for HDRS-17 and 20.05 for HAMA. The detailed demographic and clinical characteristics for participants were displayed in **Table 1**.

**Table 1:** Demographic and clinical characteristics of participants enrolled for *TRANK1* mRNA test (mean ± SD).

Items	BD (n = 22)	HC (n=28)	*P*
Gender, female (%)	13 (59.1%)	17 (60.7%)	0.118 ^#^
Age, year-old	19.45 ± 5.69	21.46 ± 1.23	0.907^Δ^
BMI, kg/m^2^	22.35 ± 5.71	21.33 ± 3.09	0.642^Δ^
Right-handed, %	100%	100%	1
HDRS-17	26.64 ± 11.05	–	–
HAMA	20.05 ± 8.65	–	–
YMRS	3.55 ± 2.34	–	–

BD, bipolar disorder; HC, healthy control; SD, standard deviation; HDRS-17, 17-item Hamilton Depression Rating Scale; HAMA, Hamilton Anxiety Rating Scale; YMRS, Young Mania Rating Scale. ^#^Pearson chi-square test; ^Δ^Independent sample t test.

### Elevated Serum *TRANK1* mRNA Expression in Untreated BD Patients

**Figure 1:**
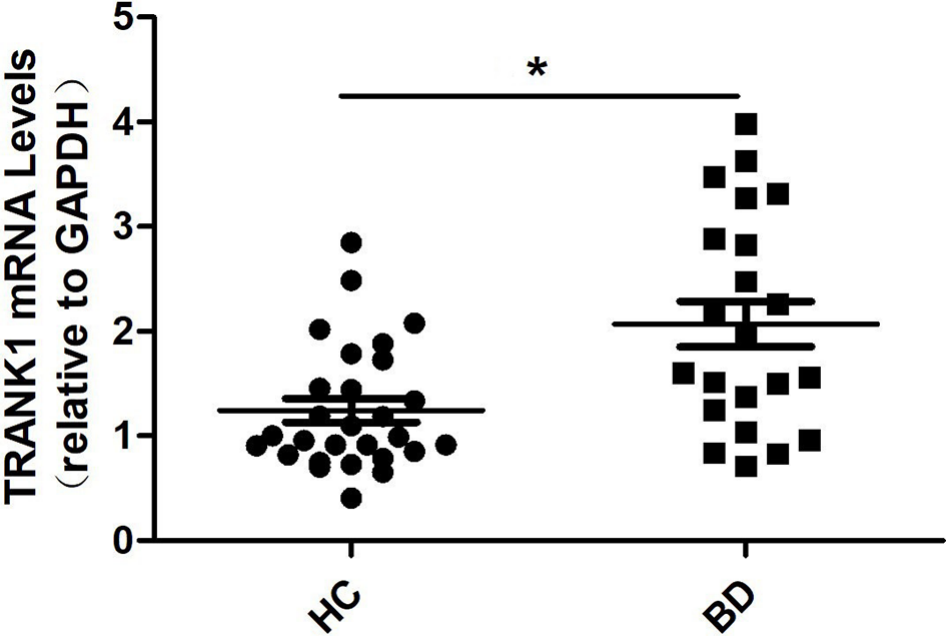
Peripheral blood levels of *TRANK1* mRNA by qRT-PCR in patients with a depressive episode of BD (n = 22) and healthy controls (n = 28). *p = 0.002.

Compared to HCs, patients with BD depression showed an elevated level of *TRANK1* mRNA in peripheral blood (*P* = 0.002, **Figure 1**).

### Depression-Like Behavior in “BD Microbiota” Recipient Mice

**Figure 2:**
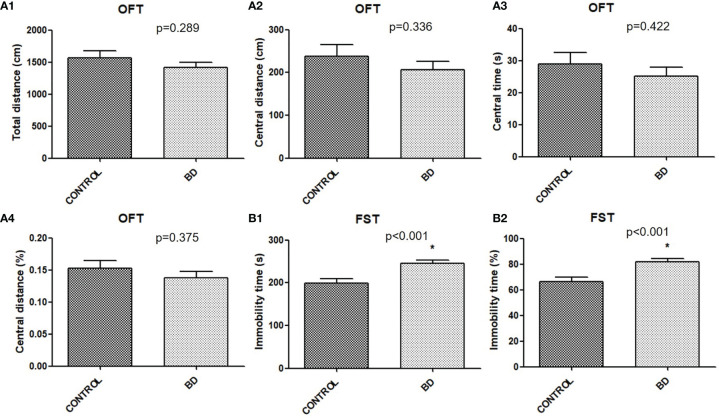
Behavioral consequences of mice transplanted with fecal microbiota from untreated BD patients or healthy controls (BD mice, n=40; control mice, n=22). **(A)** OFT. The total distance (cm), central distance (cm), central time (s) and central time percentage (%), were measured (A1-A4). **(B)**FST. The immobility time (s), and immobility time percentage (%), were measured (B1, B2). All data were presented as means ± SEM. *p < 0.001 using independent t tests. FST, forced swimming test; OFT, open field test.

Mice were transplanted with feces from either unmedicated patients with BD depression or healthy controls to verify behavioral consequences. Although no significant differences were found in OFT central distance (*P* = 0.289), central distance (*P* = 0.336), central time (*P* = 0.422) or percentage (*P* = 0.375) between the two groups (**Figure 2**), FST immobility time (*P* < 0.001) and percentage (*P* < 0.001) were significantly increased in “BD microbiota” recipient mice than “healthy microbiota” recipient mice (**Figure 2**).

### Activated Neuroinflammation and Enhanced Expression of TRANK1 mRNA in Mice Brain After FMT

**Figure 3:**
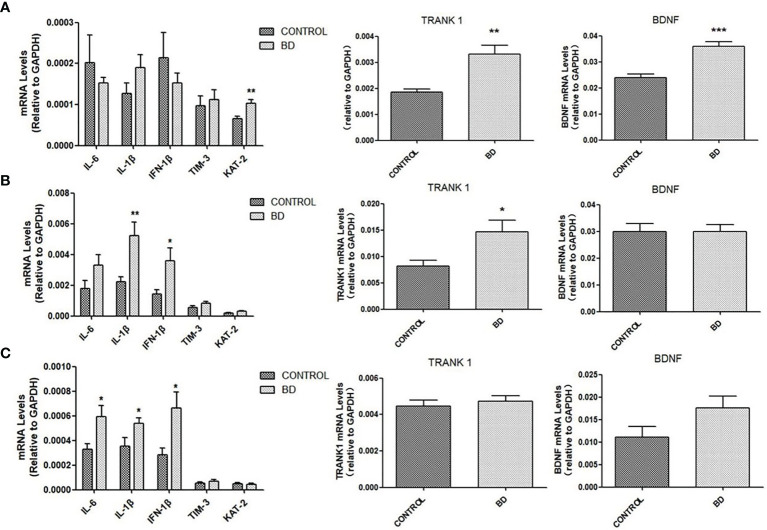
mRNA levels of IL-6, IL-1β, IFN-1β, TIM-3, KAT-2, *TRANK1* and BDNF measured by qRT-PCR in hippocampus **(A)**, prefrontal cortex **(B)** and corpus striatum **(C)** of mice colonized with fecal microbiota from healthy controls or untreated patients with a depressive episode of BD (n=10 mice in each group) at the 4th week after fecal microbiota transplantation. All data are presented as means ± SEM. *p < 0.05, **p < 0.01, ***p < 0.001 using independent sample t-test.

We further examined the influence of FMT on brain mRNA expressions of molecules linking to neuroinflammation, neurotransmitter production and neural growth. Compared to control mice, “BD” mice exhibited increased mRNA expressions of IL-6 in the corpus striatum (*P* < 0.05), IL-1β and IFN-1β in the prefrontal cortex and corpus striatum (all *P* < 0.05), and KAT-2 in hippocampus (*P* < 0.01). Increased mRNA level of BDNF was observed in the hippocampus of BD mice (*P* < 0.001). Of particular note, *TRANK1* mRNA expression in the hippocampus (*P* < 0.01) and prefrontal cortex (*P* < 0.05) was elevated in “BD” mice (**Figure 3**).

### LPS Treatment Activates Neuroinflammation and Upregulates TRANK1 Expression in Neurons

**Figure 4:**
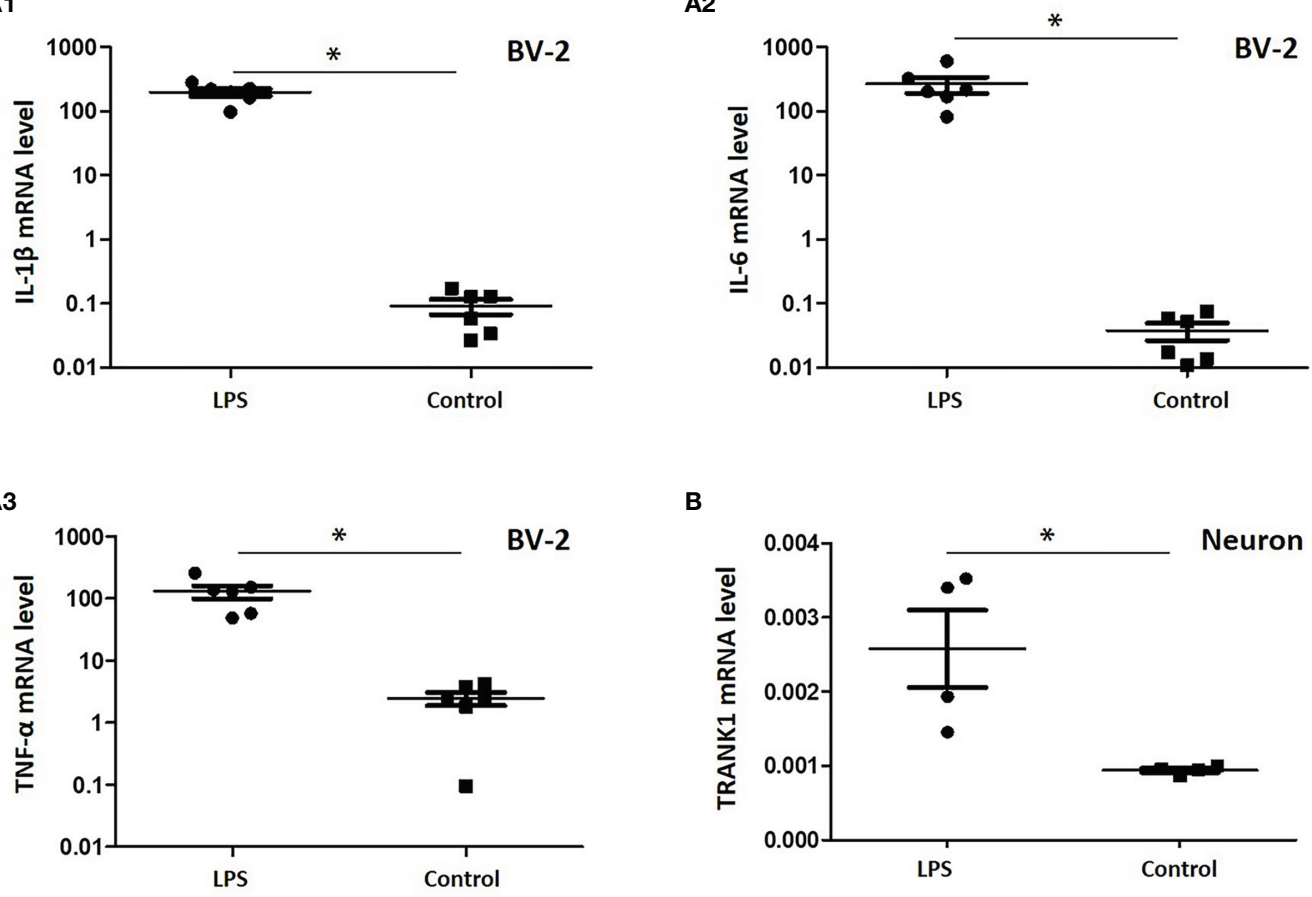
Expression of proinflammatory factors in BV-2 cells treated with LPS and the effects of neuroinflammation on TRANK1 expression in neurons. **(A)** A1, A2, A3 represented the mRNA levels of IL-1β, IL-6 and TNF-α, respectively; **(B)** The effects of LPS-treated BV-2 supernatant on the expression of TRANK1 mRNA in neurons. *p < 0.05 using independent sample t-test.

To verify the effects of gut microbe-derived components on microglia and neurons, we performed a study *in vitro* with LPS, a typical component of gram-negative bacteria. Following LPS treatment, the mRNA levels of proinflammatory factors in BV-2 cells, including IL-1β, IL-6 and TNF-α, were significantly promoted (all *P* < 0.05, **Figure 4A**). When stimulated by LPS-treated BV-2 cell supernatant, hippocampus neurons of rats displayed an increased level of *TRANK1* mRNA (*P* < 0.05, **Figure 4B**).

### Overexpression of *TRANK1* in Neurons Leads to Decreased Spine Density

**Figure 5:**
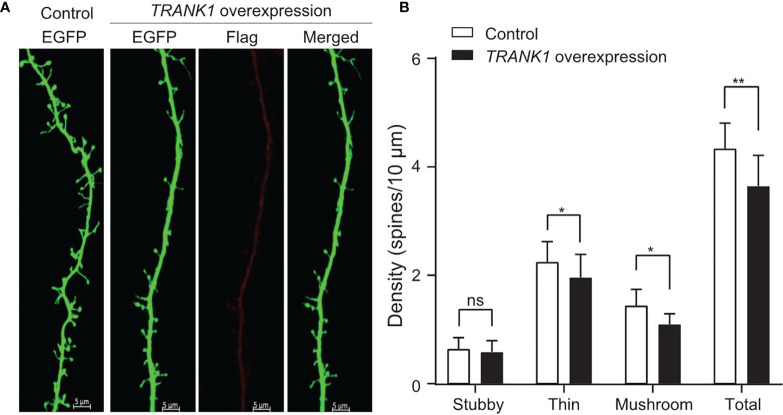
*TRANK1* overexpression decreases dendritic spine density in rat primary cortical neurons. **(A)** Representative microphotographs of dendrites from neurons transfected with either control or *TRANK1* overexpression vectors plus the Venus vector (which encodes EGFP protein) at DIV14. Scale bar: 5 µm. **(B)** Density of total, stubby-shaped, thin-shaped, and mushroom-shaped spines in neurons overexpressing *TRANK1* compared with control neurons. Dendritic spine density was quantified as the number of spines normalized to 10 μm of dendritic length (**p < 0.001, *p < 0.01, n=15/16 neurons per condition). ns, not significant.

Our results indicated that higher*TRANK1* expression was possibly linked to depressive episodes in BD. To identify whether the increased expression of *TRANK1* causes any functional consequences, we examined its effects on the morphology and density of dendritic spines, a potential endophenotype for BD [43]. Primary cortical neurons transfected with *TRANK1*-overexpression vector exhibited significantly altered densities of dendritic spines compared with those transfected with the control vector (**Figure 5A**). In brief, the density of total dendritic spines significantly decreased in neurons overexpressing *TRANK1* (*P* < 0.001, two-way t-test, **Figure 5B**), probably due to the significant reduction of the densities of thin and mushroom spines compared with neurons in the control group (both *P* < 0.01, two-way ANOVA, **Figure 5B**), whereas stubby spine did not alter obviously. This result is consistent with the previously reported decrease of dendritic spine density in the postmortem brains of BD patients (27), suggesting that *TRANK1* might participate in the pathogenesis of BD (depressive episodes) *via* modulating this pivotal physiological feature.

## Discussion

In the current study, an upregulated circulating *TRANK1* expression was firstly reported in patients with BD depression. Accumulating large-scale GWAS studies have provided adequate evidence that *TRANK1* is a robust susceptible gene of BD (3–6). Our finding further supports the involvement of *TRANK1* in the BD pathogenesis. The FMT findings in our study revealed that fecal microbiota from patients with BD depression were sufficient to elicit depression-like behavior in mice, which may link to activated neuroinflammation and an elevated expression of *TRANK1*. It is noteworthy that *TRANK1* overexpression resulted in decreased dendritic density in primary neurons, which may comprise neuronal morphological basis of depressive episodes in BD.

In recent years, the implications of the microbiota-gut-brain axis in the neuropsychiatric disorders have been gradually uncovered (28). The use of prebiotics, probiotics and FMT as adjuvant therapies for mentally ill patients is becoming a research hotspot (28). Some researchers even have proposed the study protocol for verifying the efficacy and safety of FMT in bipolar patients during depressive episodes (29). Nonetheless, most of the explorations are still limited to animal experiments. Previous studies have shown gut microbiota from mentally ill patients were sufficient to elicit disease-specific behavioral patterns in recipient mice (30–33). However, no study has ever explored this phenomenon in BD patients. To address the effects of BD microbiota on brain function and behavior, “BD microbiota” or “healthy microbiota” recipient mice were designed to observe the behavioral consequences in our study. Compared to “healthy microbiota” recipient mice, increased immobility time in FST was observed in “BD microbiota” recipient mice, which was interpreted as depression-like behaviors.

To further determine the brain molecular mechanisms underlying the behavioral changes, mRNA expression levels of inflammatory factors, BDNF, KAT-2 and *TRANK1* were measured in the hippocampus, prefrontal cortex and corpus striatum of recipient mice. We found an up-regulation of immune mediators, such as IL-6, IL-1β and IFN-1β. Neuroinflammation is recently considered to be involved in the pathogenesis of major psychiatric disorders, including schizophrenia, BD and major depressive disorder (MDD) (34–36). Similarities in the pattern of cytokine networks indicate a common underlying immune dysfunction of these psychiatric disorders (36). Although no inflammatory biomarker was independently capable of differentiating mood phases of BD, a combination of high-sensitivity C-reactive protein/IL-6, BDNF/TNF-α or soluble TNF-α receptor 1 was identified to be mood phase-specific in BD (37). In our study, an increased level of IL-6 and IL-1β indicated the activation of neuroinflammation due to fecal microbiota transplantation from patients with a depressive episode of BD. The actions of IFN-1β, however, include induction of regulatory mediators, decreasing the secretion of proinflammatory cytokines and modulating cell trafficking across the blood–brain barrier (BBB) (38). Therefore, a parallel activation of both proinflammatory and immuno-suppressive processes reflect a compensatory mechanism of neuroinflammation in BD. In addition, increased mRNA levels of BDNF and KAT-2 were detected in “BD microbiota” recipient mice. BDNF is the dominant neurotrophin in brain, and the BDNF/TrkB signaling pathway plays an important role in synaptic maturation, plasticity, neuronal growth and survival (39). Available findings in relevant to peripheral BDNF levels amongst BD patients were inconsistent (40–42). In particular, longer duration of illness was associated with high serum BDNF level (40). Acute stress treatment may upregulate the expression of hippocampal BDNF mRNA (43). Therefore, increased hippocampal BDNF transcripts in recipient mice were possibly caused by the acute colonization of “BD microbiota”. However, some researchers pointed out that the neurodevelopmental trait of BDNF could be attenuated by the underlying neuroinflammation processes (42). KAT-2 is a key enzyme catalyzing the synthesis of KYNA from KYN, which was known as the tryptophan metabolic pathway (44). The tryptophan catabolism can be activated upon inflammatory stimuli, such as viral invasion, bacterial LPS, and IFN stimulation (45, 46). Therefore, in “BD microbiota” recipient mice, CNS inflammatory status caused by LPS-induced immune activation and enhanced IFN-1β stimulation, eventually promoted the expression of KAT-2 and increased the concentration of KYNA. Abnormal KYNA level is implicated in various neuropsychiatric disorders, including schizophrenia (31, 44), Alzheimer’s disease and other illnesses (44). In patients with BD, an increased CSF level of KYNA was also reported in previous studies and was associated with manic episodes and psychotic features (47, 48). These findings indicated the dysregulation of KYN-KYNA metabolic pathway was possibly linked to BD pathophysiology.

The interplay between gut microbiota and host genes expression has been rarely investigated in the microbiota-gut-brain regulation. Inspiringly, our study is the first to report that gut microbiota can influence the CNS expression of *TRANK1* in the hippocampus and prefrontal cortex. Expression of TRANK1 was observed under different pathological conditions, such as neuroinflammation, disrupted formation and functioning of blood vessels in the BBB (49). In a rat model of psychosis, one-week social isolation led to increased mRNA expression of *TRANK1* in the prefrontal cortex and other specific genes involved in neuroinflammation, formation and integrity of the BBB, and cerebral blood vessel morphogenesis (49). This is consistent with our findings that CNS inflammatory profiles are elevated in “BD microbiota” recipient mice. Moreover, gut microbial dysbiosis was associated with impaired integrity and increased permeability of the BBB (50, 51). Loss of BBB integrity was implicated in neuropsychiatric diseases, such as depression, bipolar disorder and schizophrenia (51). A compromised BBB may facilitate the entrance of gut microbes-derived components, such as LPS, into the CNS and activate the neuroinflammation. In accordance with previous studies (52), we found that *in vitro* LPS stimuli could promote the transformation of microglia into the M1 phenotype and the secretion of proinflammatory factors (IL-1, IL-6 and TNF-α), which was capable of upregulating the expression of *TRANK1* in neurons. Furthermore, we showed that overexpression of *TRANK1* resulted in loss of dendritic spine density in cortical primary neurons. In patients with severe mental illnesses, dendritic spine abnormalities in the prefrontal cortex have been considered to be neurobiological mechanisms underlying these diseases (53, 54). Impairment of the dendritic spine development could lead to cognitive deficits and behavioral abnormalities in BD (53, 55). Deficits in dendritic spine morphogenesis link to synaptic dysfunction and synapse loss (56). In other words, overexpression of *TRANK1* is possibly associated with brain dysfunction by hampering the growth of dendritic spines and disrupting synaptic functions.

Several limitations in this study need to be mentioned. First, although we found that circulating *TRANK1* mRNA level was elevated in patients with BD depression, no patients with manic episodes or other major psychiatric disorders, such as schizophrenia and MDD, were included in this study. Therefore, we cannot conclude that the upregulated expression of *TRANK1* was an exclusive trait of BD depression. Another weakness of our study is the absence of manic/hypomanic and remissive participants, we thus are unable to compare the impacts of different conditions on the gut microbiota, as well as the behavioral patterns following FMT. Third, lack of examining the gut microbiota in mice weakened the stringency of this study, though different conditions in the mice brain existed. Fourth, although we observed a regulatory role of LPS-induced pro-inflammatory environment on *TRANK1*, further explorations *via* inhibiting the LPS-toll-like receptors interactions and its effects on *TRANK1* expression are also needed.

Taken together, the current study revealed an upregulated circulating *TRANK1* expression in patients with BD depression and a potential regulatory role of gut microbiota on neuroinflammation and *TRANK1* expression in the brain. Based on the gene × microbiota perspective, our study helps to better understand the role of *TRANK1* in the BD pathogenesis and its involvement in the gut-microbiota-brain regulation.

## Data Availability Statement

The raw data supporting the conclusions of this article will be made available by the authors, without undue reservation.

## Ethics Statement

The studies involving human participants were reviewed and approved by Institutional Review Board of the First Affiliated Hospital, Zhejiang University School of Medicine. Written informed consent to participate in this study was provided by the participants’ legal guardian/next of kin. The animal study was reviewed and approved by Animal Ethical Committee of First Affiliated Hospital, Zhejiang University School of Medicine.

## Author Contributions

Designed the experiments: SH. Collected the clinical samples: JL, PFZ, CX, LW, XG, DZ, YC, and HH. Performed the animal and in vitro cell study: PFZ, ML, JJ, YL, HL, XC, and PZ. Performed the analysis and drafted the manuscript: JL, PZF, JJ, and TM. Revised the manuscript for intellectual content: SH. All authors contributed to the article and approved the submitted version.

## Funding

This study was granted by the National Natural Science Foundation of China (81971271), and the Natural Science Foundation of Zhejiang Province (LQ20H090013). The funding agencies had no role in the design and conduct of the study; collection, management, analysis, and interpretation of the data; preparation, review, or approval of the manuscript; and decision to submit the manuscript for publication.

## Conflict of Interest

The authors declare that the research was conducted in the absence of any commercial or financial relationships that could be construed as a potential conflict of interest.

## Publisher’s Note

All claims expressed in this article are solely those of the authors and do not necessarily represent those of their affiliated organizations, or those of the publisher, the editors and the reviewers. Any product that may be evaluated in this article, or claim that may be made by its manufacturer, is not guaranteed or endorsed by the publisher.
