# Supplementary material for: New Evidence of Gut Microbiota Involvement in the Neuropathogenesis of Bipolar Depression by TRANK1 Modulation: Joint Clinical and Animal Data
Source: Front Immunol. 2021 Dec 21;12:789647. doi: 10.3389/fimmu.2021.789647 (PMC8724122; doi:10.3389/fimmu.2021.789647)
Supplement: Supplementary file 1 [file DataSheet_1.docx]

**Supplemental materials**

**Supplemental Table 1.** Demographic and clinical characteristics of subjects enrolled for fecal transplantation (mean ± SD).

**Supplemental Table 2.** Primers used for genes of interest in mice brain.

**Supplemental Table 3.** Primers used for genes of interest in BV-2 cells and neurons in vitro.

**Supplemental Table 1.** Demographic and clinical characteristics of subjects enrolled for fecal transplantation (mean ± SD)

| Items | BD (n = 10) | HC (n=10) | t/χ^2^ | *P* |
| --- | --- | --- | --- | --- |
| Gender, female (%) | 6 (60%) | 4 (40%) | 0.800 | 0.371 |
| Age, year-old | 26.10 ± 7.48 | 27.00 ± 9.17 | -0.241 | 0.813 |
| Height, cm | 167.60 ± 8.81 | 166.10 ± 8.62 | -0.865 | 0.399 |
| Weight, kg | 63.91 ± 12.99 | 60.37 ± 11.60 | 0.385 | 0.705 |
| BMI, kg/m^2^ | 21.79 ± 3.38 | 22.68 ± 3.47 | -0.125 | 0.902 |
| Education, years | 12.70 ± 3.02 | 13.80 ± 2.66 | -0.578 | 0.570 |
| Age at onset, year-old | 20.40 ± 4.74 | - | - | - |
| Illness course, years | 3.75 ± 5.56 | - | - | - |
| Baseline HDRS-17 | 30.80 ± 7.50 | - | - | - |

Abbreviations: BD, bipolar disorder; HC, healthy controls; BMI, body mass index; HDRS-17, Hamilton Depression Rating Scale-17 items.

**Supplemental Table 2.** Primers used for genes of interest in mice brain

| Gene | Forward Primer (5′–3′) | Reverse Primer (5′–3′) |
| --- | --- | --- |
| GAPDH | AGGTCGGTGTGAACGGATTTG | TGTAGACCATGTAGTTGAGGTCA |
| IL-1β | ATGGCAACTGTTCCTGAACTCAACT | CAGGACAGGTATAGATTCTTTCCTTT |
| IL-6 | TCCAGTTGCCTTCTTGGGAC | GTGTAATTAAGCCTCCGACTTG |
| IFN-1β | CAGCTCCAAGAAAGGACGAAC | GGCAGTGTAACTCTTCTGCAT |
| TIM-3 | TCAGGTCTTACCCTCAACTGTG | GGGCAGATAGGCATTTTTACCA |
| KAT-2 | ATGAATTACTCACGGTTCCTCAC | AACATGCTCGGGTTTGGAGAT |
| TRANK1 | CCGCTTCTCTTCCGCCACTTC | CACCACCTTCAGCAGCCTCTTG |
| BDNF | TCATACTTCGGTTGCATGAAGG | AGACCTCTCGAACCTGCCC |

**Supplemental Table 3.** Primers used for genes of interest in BV-2 cells and neurons in vitro

| Gene | Forward Primer (5′–3′) | Reverse Primer (5′–3′) |
| --- | --- | --- |
| β-actin | CTACCTCATGAAGATCCTGACC | CACAGCTTCTCTTTGATGTCAC |
| IL-1β | GGCAACTGTTCCTGAACTCAACTG | CCATTGAGGTGGAGAGCTTTCAGC |
| IL-6 | ACAAAGCCAGAGTCCTTCAGAG | CATTGGAAATTGGGGTAGGA |
| TNF-α | GTCTACTGAACTTCGGGGTGAT | ATGATCTGAGTGTGAGGGTCTG |
| TRANK1 | ACAGAGGCGGAGAAGAGCATAGAG | CCGTGGCTGTCATGTCGTTGAG |
